# Accrual to a randomised trial of adjuvant whole brain radiotherapy for treatment of melanoma brain metastases is feasible

**DOI:** 10.1186/1756-0500-7-412

**Published:** 2014-06-30

**Authors:** Gerald B Fogarty, Angela Hong, Kari Dolven Jacobsen, Claudius H Reisse, Brindha Shivalingam, Bryan Burmeister, Lauren E Haydu, Elizabeth Paton, John F Thompson

**Affiliations:** 1 Melanoma Institute Australia, Sydney, Australia; 2 Sydney Medical School, The University of Sydney, Sydney, Australia; 3 Oslo University Hospital HF, The Norwegian Radium Hospital, Oslo, Norway; 4 Australia and New Zealand Melanoma Trials Group (ANZMTG), North Sydney, Australia; 5 Princess Alexandra Hospital, Brisbane, Australia; 6 Trans - Tasman Radiation Oncology Group (TROG), Newcastle, Australia

**Keywords:** Radiotherapy, Metastases, Melanoma, Brain, Trial, Pilot project, Feasibility, Whole brain radiotherapy, Randomised trial

## Abstract

**Background:**

Brain metastases (BMs) are common in melanoma patients. Adjuvant whole brain radiotherapy (WBRT) following local treatment of intracranial melanoma metastases with neurosurgery and/or stereotactic radiosurgery is controversial. A randomised trial is needed. However, accrual to WBRT trials has been problematic. A pilot study by Australia and New Zealand Melanoma Trials Group (ANZMTG) was conducted to see if accrual was feasible.

**Methods:**

Sites canvassed for interest included those who treat melanoma patients, had a proven accrual in previous melanoma trials and who had the relevant infrastructure support. Feasibility forecasts from interested sites were sought. These were compared to the patient numbers documented in the research contracts. A target accrual of 60 patients in 2 years was set. Funding was sought for the pilot study. Basic demographics of the pilot study cohort were collected.

**Results:**

The first centre opened December 2008; the first patient was randomised in April 2009. The pilot accruing period concluded in September, 2011. In 30 months, 54 patients from 10 of a total of 17 activated sites in Australia (39, 72%) and in Norway (15, 28%) had been accrued. Feasibility forecasts predicted 133 trial eligible patients per year (including 108 Australian + 25 International patients). Site estimates generally overestimated accrual with 4 of 17 active sites estimating within 50% of target numbers. Sites with patient estimates calculated from records were more accurate than those estimated from memory. The overall recruitment target was lower in the research contracts when compared to the feasibility evaluation. Basic demographics of the pilot study revealed 62% of patients were males; 58% had a single metastasis, 28% had two and 14% had three metastases. 12-month overall survival was 50%.

**Conclusions:**

Despite only 54 patients and not the full 60 patient target being accrued in two years the Trial Management Committee and Data Safely Monitoring Committee approved the continuation of the pilot study to the main trial. On the basis of this successful pilot study, funding was achieved for the full study. 143 patients of a target of 200 have been randomised by June 2014.

## Background

Brain metastases (BMs) are common in patients with metastatic melanoma [1,2] and are the cause of death in the majority of them [2,3]. Whole brain radiotherapy (WBRT) is a common adjuvant treatment after local treatment of BMs with neurosurgery and/or stereotactic radiation (SR). However, there is no level one evidence to support this approach in melanoma. Proponents say that WBRT improves palliation by prolonging intracerebral control [4]. Opponents say that WBRT does not increase survival, may cause neurocognitive problems and may not prevent intracerebral progression [5,6]. A randomised controlled trial (RCT) is needed to resolve this controversy.

Based on trials of WBRT in BMs of all histologies, an accrual target of two hundred patients for the RCT was considered necessary, and a protocol was developed [7]. However, accrual to previous WBRT trials has been problematic. Trials of WBRT for BMs in all histologies have taken many years to accrue [8-10]. Some have even closed prematurely [11,12] (Table 1). The majority of these trials also had no or inappropriate tests for neurocognitive function (NCF), an important endpoint in this scenario. Meaningful NCF testing takes time and requires expertise, and therefore has funding implications.

**Table 1:** Accrual to whole brain radiotherapy trials

**Trial**	**Design**	**Histology and no. of BMs**	**Appropriate NCF testing**	**Actual recruitment (patients/years)**	**Average no. of patients accrued per year**	**Total number of melanoma patients (%)**
Patchell et al. [8]	WBRT vs. OBS	All solid tumours (mainly lung) Sol BM post- surgery	No	96/8 years	12	2 (2%)
Aoyama et al. [9]	SR vs. WBRT + SRS	All solid tumours (mainly lung) 1–4 BMs	No	132/4 years	33	Unknown
Kocher et al. [10]	SR vs. SR + WBRT	All solid tumours (mainly lung) 1–3 BMs	No	359/11 years	33	18 (5%)
Chang et al. [11]	SR vs. WBRT + SR	All solid tumours (mainly lung) 1–3 BMs	Yes	58/6 years	10 Early closure	7 patients (12%)
Roos et al. [12]*	WBRT vs. OBS	All solid tumours sol BM post-SR/surgery	No	19/3 years	6	3 (16%)
This study	WBRT vs. observation	Melanoma only 1–3 BMs post-SR/surgery	Yes	54/2 years	27	54 (100%)

*WBRT* - Whole Brain Radiotherapy.

*SR* - Stereotactic Radiosurgery.

*Sol BM* - solitary Brain Metastases.

*OBS*- observation.

*NCF* - Neurocognitive.

*Early closure due to poor accrual.

In order to ensure that a RCT of WBRT in melanoma could be completed, a pilot study was designed. The purpose of the study was to assess whether accrual to the main trial was feasible. The pilot study was designed to flow on to the main trial, so that if the pilot was successful, the patients accrued would automatically become part of the main study.

## Methods

This trial is an international multi-centre, open-label, stratified, 2-arm randomised phase III trial. The trial has been approved by the Cancer Institute NSW Clinical Research Ethics Committee #2007C/11/032 and relevant hospital ethics committees in each participating centre. Written informed consent for participation in the study was obtained from participants.

Initially, attention during the pilot study was aimed at finding sites interested in contributing patients and canvassing their opinion as to whether this was a trial that they considered worthwhile.

### Sites

Sites canvassed included those with a proven accrual record in previous melanoma trials; were sites that treated patients with melanoma brain metastases; were sites that had available neurosurgery and/or SR, were sites adequately resourced to engage in research; and had functioning melanoma multidisciplinary clinics.

### Feasibility forecasts

Feasibility forecasts from interested sites were sought. From this information, and with the knowledge of the difficulty of accrual to WBRT trials of all histologies, and the needs of funding agencies, a target accrual of 60 patients in 2 years was set for the pilot study. This time frame took into account the customary slow start up time for new trials, which includes time for administrative arrangements to be made and for staff education.

Sites were then encouraged and assisted to open the trial in a timely fashion. Activated sites were also required to sign a research contract to accrue to the trial as part of the usual trial governance. This contract also asked for a prediction of recruitment. The numbers predicted in the feasibility forecasts were able to be compared with those predicted in the research contract. The success of the study was to be judged by the Trial Management Committee. However, the real test of whether the study was successful would be whether accrual to the pilot study would convince the funding authority to allocate sufficient funds for the main trial.

### Funding

Funding from the Australia and New Zealand Melanoma Trials Group (ANZMTG), Trans-Tasman Radiation Oncology Group (TROG), The University of Sydney and the Australian Government’s National Health and Medical Research Council (NHMRC) was sought for the pilot study.

## Results

**Figure 1:**
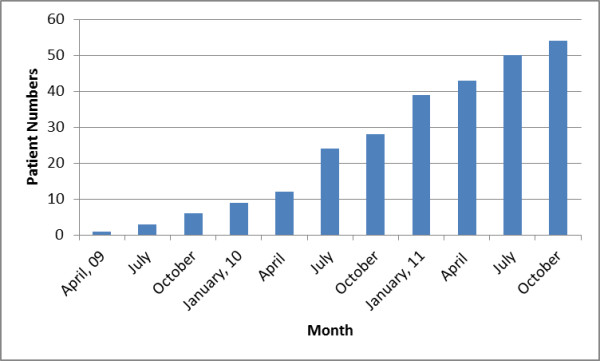
Pilot study patient recruitment over time – April ’09-October ’11.

The first recruiting centre was opened in December 2008, with the first trial patient being randomised in April 2009. The pilot grant reporting period concluded on 30th June 2011. Accrual increased proportionately as more centres opened. In 30 months, 54 patients had been accrued from 10 of a total of 17 activated sites in Australia and in Norway by the conclusion of the pilot study (Tables 2 and 3; Figure 1). Australian sites contributed 39 (72%) of the 54 recruited patients.

**Table 2:** Pilot patient recruitment by location

**State/country**	**Recruitment up to September 2011**
New South Wales	24
Queensland	11
Victoria	3
South Australia	1
Western Australia	0
International	15
**Total**	**54**

**Table 3:** Pilot patient recruitment over time

**Year**	**Month**	**Patients**
2009	April	1
	July	3
	October	6
2010	January	9
	April	12
	July	24
	October	28
2011	January	39
	April	43
	July	50
	October	54

### Sites

In total 29 sites internationally were approached and there were 19 positive responses received. All sites that responded positively reported having appropriate knowledge and clinical trials experience including International Congress of Harmonization and Good Clinical Practice (ICHGCP) training.

### Feasibility forecasts

Feasibility forecasts of the 19 possible major melanoma treatment centres is summarised in Table 4. Fourteen responses came from radiation oncologists, with three responses from neurosurgeons and two responses from medical oncologists. Nineteen sites, including two international sites, predicted a total of 133 trial eligible patients per year (including 108 Australian + 25 international patients). Two problematic logistic issues were reported at that time. Two sites reported that they did not have adequate access to radiotherapy and imaging services mandated by the study protocol. They were unable to proceed with the study, leaving 17 sites able to proceed.

**Table 4:** **Pilot study** - **site actual accrual versus feasibility forecasts and research contract estimates**

**Site Code**	**State/country**	**How was the patient estimate was calculated**	**Number of patients estimated in the feasibility forecast**	**Number of patients estimated in the research contract**	**Was number of patients in research contract less than what was estimated in the Feasibility Forecast?**	**Actual patients accrued to study**	**Was accrual at the site within 50% of what was specified in the research contract?**
1^#^	NOR	NA	10-15	5	Yes	15	Yes
2^*^	NSW	Records	5	5	No	20	Yes
3^^^	NSW	Memory	12	4	Yes	2	No
4^*^	NSW	Memory	5-6	6	No	0	No
5^*^	NSW	Memory	5-6	2	Yes	0	No
6^^^	NSW	Records	6	2	Yes	1	No
7^*^	NSW	Records	3	2	Yes	0	No
8^*^	NSW	Memory	8	4	Yes	1	No
9^*^	NSW	Memory	4	NS	NA	0	No
10^*^	VIC	Memory	20	10	Yes	2	No
11^*^	VIC	Records	10	5	Yes	1	No
12^*^	QLD	Records	2-3	2	Yes	3	Yes
13^*^	QLD	Memory	10	10	No	8	Yes
14^*^	QLD	Records	2-3	2	Yes	0	No
15^*^	QLD	Records	2	2	No	0	No
16^*^	SA	N/A	NA	2	NA	1	No
17^*^	WA	N/A	NA	NS	No	0	No
18^^^	VIC	N/A	10	NA	NA	NA	NA
19^#^	Europe	N/A	10	NA	NA	NA	NA
**Total**			**124**-**133**	**63**	**10**	**54**	**4**/**17 Yes**

^#^Department of Medical Oncology.

*Department of Radiation Oncology.

^^^Department of Neurosurgery.

*NOR* – Norway; NSW – New South Wales; *AU* – Australia; *VIC* – Victoria; *QLD* – Queensland; *SA* – South Australia; *WA* – Western Australia; *NA* – not applicable; *NS* - Not specified.

Site feasibility forecasts generally overestimated possible accrual. Only four of seventeen activated sites recruited the patient number (to within 50%) predicted in their feasibility forecast during the pilot study accrual period. Seven of nineteen sites referred to their medical records to provide their feasibility numbers, the remaining sites provided feasibility numbers from memory or did not specify how they arrived at their numbers. Sites that reported patient estimates by referring to their medical records were subsequently found to be more accurate in predicting accrual than those that did not.

The numbers predicted in the feasibility forecasts were compared with those predicted in the research contract. Ten of the seventeen activated sites reduced the number of estimated trial patients from feasibility forecasts to research contracts. Of these 10 sites, two sites were successful in achieving the revised target accrual, five sites randomised some patients but less than specified in their contracts, and three sites failed to randomise any patients to the protocol in the accrual period.

### Funding

The initial funding application for the pilot study was successful, which made the whole project possible. In 2006, the ANZMTG and TROG Executive and Scientific Committees approved protocol development, and preliminary seed funds ($AU18,000) were allocated in support of the protocol development activities. In 2007, ANZMTG successfully applied to the Commonwealth of Australia Department of Health and Aging for support of the pilot study ($AU281,019). Due to delays in activating sites, a 12 month extension was granted until 30 June 2011.

### Demographics

The pilot patient demographic information is presented (Table 5). Baseline analysis of this population demographic showed even distribution between the treatment groups (27 patients per arm). The pilot cohort included 62% males and 38% females (n = 54). The age of patients ranged from 26 to 83 years; with the mean age of Australian patients being slightly older than international patients (61 years vs 56 years). Mean age was relatively balanced between treatment arms (WBRT 60 years vs Observation 58 years). The majority of patients in the pilot cohort presented with a solitary brain metastasis (58%), and the remainder with two metastases (28%) or with three metastases (14%). A minority (33%) of pilot trial patients presented at the baseline visit with no evidence of extracranial disease present.

**Table 5:** Pilot study patient demographics (54 patients)

**Site**	**Number of participants**	**%**
Australian	39	72
International	15	27
Male	34	62
Female	20	37
Age (years)	Mean 61 (AU) Mean 56 (Norway) Range: 26 to 83	
Local treatment of BMs		
Neurosurgery	45	83
Stereotactic	8	15
None	1	2
Patient presentation at the baseline visit		
1 BM	31	57
2 BM	15	27
3 BM	8	14
Extracranial disease – present	36	66
Extracranial disease - absent	18	33

*AU* - Australia.

*BMs* – Brain metastases.

**Figure 2:**
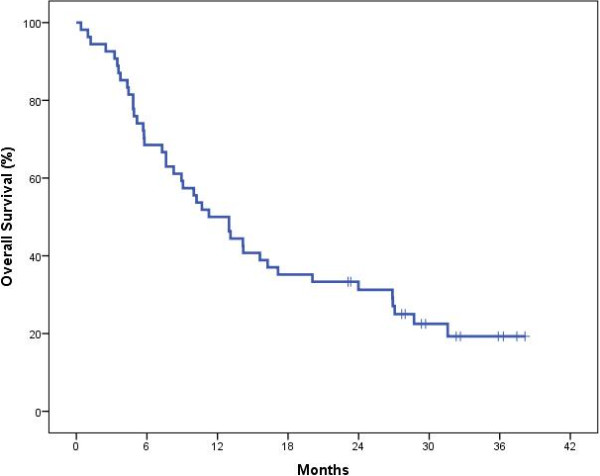
Overall survival curve for pilot study patients (N = 54).

Cumulative overall survival (Table 6; Figure 2) for this pilot cohort was slightly higher than otherwise reported for these patients [2]. The study had 6-month overall survival of 68.5% and 12-month overall survival of 50.0%. There were no trial related significant adverse events. Data audit after the study was competed revealed that data quality was high, especially with completion of NCF and Quality of Life assessments.

**Table 6:** Pilot study overall survival from randomisation

**Months**	**Percentage surviving**
6	68.5
12	50.0
18	35.2
24	31.3

## Discussion

RCTs are fundamental to progressing evidence–based medicine. Vibrant trial accrual is essential for timely RCT completion. Accrual has been difficult in some RCT scenarios, for example, WBRT trials of all histologies. Accrual for a WBRT RCT for a single histology such as melanoma was thought likely to be even more difficult.

In our pilot study, 54 patients out of a projected 60 were recruited to the study from 10 of 17 activated sites. Australian sites contributed 39 (72%) of the 54 recruited patients. International sites were important contributors to the pilot study. Despite only 54 patients and not the full 60 patient target being accrued in two years the Trial Management Committee and Data Safely Monitoring Committee approved the continuation of the pilot study to the main trial.

### Sites

Successful strategies included selecting sites with investigators having proven accrual in previous melanoma trials. Another successful strategy was the ANZMTG coordinating office providing centralised site support and assisting with study start-up activities (assisting and coordinating central and local ethics submissions) which directly accelerated the start of the study at the individual sites.

### Feasibility forecasts

More radiation oncologists completed the feasibilities than any other clinical speciality, which was consistent with this being a radiotherapy trial question. As in many clinical trials, overall recruitment was lower than predicted by the sites. Site estimates generally overestimated possible accrual with only 4 of 17 sites accurate to within 50% of target estimates. Sites gathering data from records gave more accurate estimates of feasibility than sites estimating from memory. Perhaps central bodies should insist on data only from medical records when assessing sites for inclusion in trials.

Sites in general decreased the expected number of patients to be randomised when their feasibility forecast was compared with the research contracts. The reasons for this are not clear but may reflect an increasing awareness of the difficulty of accrual as the site became more involved in the trial. In general, the actual number of patients recruited was more in agreement with the number specified in the site research contracts than the initial feasibility forecasts.

There were still some sites which, despite agreeing to participate in the trial and having a reduced patient target in their contracts, failed to screen and randomise any trial patients. The reasons for this are also not clear. Possibly a perceived lack of clinical equipoise in respect to this trial question may be the strongest reason. Other reasons why recruitment was not optimal include ethical considerations, lack of time and interest, new systemic treatments interfering with brain metastases treatments, and perhaps conflicts between different medical specialties e.g. medical versus radiation oncology. These reasons may not have been apparent to the site investigators when completing their feasibility forecasts and then the research contracts for the study. Nevertheless the pilot recruitment compares favourably with other whole brain radiotherapy trials.

### Funding

The success with securing funding was pivotal to this study. Following the successful completion of the pilot study, an additional NHMRC grant ($AU591, 010) was applied for and awarded. In fact, all funding requests for the main trial were granted in full and no funding application has been refused. This approach of securing funding for a pilot study to assess feasibility, and then applying for funding for the full study after a successful pilot study proved to be a worthwhile strategy. The funding agencies knew that they did not have to commit to funding for a whole trial that may not complete, and this was attractive to them. The trial organizers could gain experience, for example, by focusing on increasing accrual, without being distracted by questions that may have arisen if a full trial was running.

## Conclusions

A RCT investigating WBRT after local treatment of melanoma BMs is required. However, WBRT trials are hard to accrue. A pilot study with a target of 60 patients in 2 years was undertaken. 54 patients were randomised in 30 months. International involvement and funding were essential. Only 4 of 17 sites were within 50% of their feasibility forecasts. Sites that based these forecasts on a patient record review rather than from memory were more reliable. Sites generally decreased their accrual estimates from feasibility forecasts to the research contracts. The funding agencies have now funded the full trial which is now under way, with 143 patients of the target 200 randomised to date.

## Abbreviations

ANZMTG: Australia and New Zealand Melanoma Trials Group; AU: Australia; BMs: Brain metastases; ICHGCP: International Congress of Harmonization and Good Clinical Practice; NA: Not applicable; NCF: Neurocognitive function; NHMRC: National Health and Medical Research Council; NOR: Norway; NS: Not specified; NSW: New South Wales; OBS: Observation; QLD: Queensland; RCT: Randomised controlled trial; SA: South Australia; Sol BM: Solitary Brain Metastases; SR: Stereotactic radiation; TROG: Trans-Tasman Radiation Oncology Group; VIC: Victoria; WA: Western Australia; WBRT: Whole brain radiotherapy.

## Competing interests

The authors declare that they have no competing interests.

## Authors’ contributions

GBF – concept, design, drafting manuscript and submission; AH – concept, design, drafting manuscript; KDJ - design, drafting manuscript; CHR - data collection, drafting manuscript; BS - design, drafting manuscript; BB - design, drafting manuscript; LEH – analysis, drafting manuscript; EP – data collection and interpretation, drafting manuscript; JFT - design, drafting manuscript, final approval. All authors read and approved the final manuscript.
